# Nucleobases and corresponding nucleosides display potent antiviral activities against dengue virus possibly through viral lethal mutagenesis

**DOI:** 10.1371/journal.pntd.0006421

**Published:** 2018-04-19

**Authors:** Li Qiu, Steven E. Patterson, Laurent F. Bonnac, Robert J. Geraghty

**Affiliations:** Center for Drug Design, Academic Health Center, University of Minnesota, Minneapolis, Minnesota, United States of America; International Atomic Energy Agency, AUSTRIA

## Abstract

Dengue virus affects millions of people worldwide each year. To date, there is no drug for the treatment of dengue-associated disease. Nucleosides are effective antivirals and work by inhibiting the accurate replication of the viral genome. Nucleobases offer a cheaper alternative to nucleosides for broad antiviral applications. Metabolic activation of nucleobases involves condensation with 5-phosphoribosyl-1-pyrophosphate to give the corresponding nucleoside-5’-monophosphate. This could provide an alternative to phosphorylation of a nucleoside, a step that is often rate limiting and inefficient in activation of nucleosides. We evaluated more than 30 nucleobases and corresponding nucleosides for their antiviral activity against dengue virus. Five nucleobases and two nucleosides were found to induce potent antiviral effects not previously described. Our studies further revealed that nucleobases were usually more active with a better tissue culture therapeutic index than their corresponding nucleosides. The development of viral lethal mutagenesis, an antiviral approach that takes into account the quasispecies behavior of RNA viruses, represents an exciting prospect not yet studied in the context of dengue replication. Passage of the virus in the presence of the nucleobase **3a** (T-1105) and corresponding nucleoside **3b** (T-1106), favipiravir derivatives, induced an increase in apparent mutations, indicating lethal mutagenesis as a possible antiviral mechanism. A more concerted and widespread screening of nucleobase libraries is a very promising approach to identify dengue virus inhibitors including those that may act as viral mutagens.

## Author summary

Dengue virus is a world-wide public health menace estimated to infect hundreds of millions of people per year. Vaccines to prevent dengue virus infection have had limited success due in part to the requirement to elicit effective immune responses against the four dengue serotypes. There is an urgent unmet need for anti-dengue virus therapies. Nucleosides are effective antiviral small molecules which usually work by inhibiting the accurate replication of the viral genome. Typically, nucleosides must be converted within the cell to their triphosphate form to inhibit virus replication, thus inefficient phosphorylation often leads to suboptimal activity. We screened a small library of nucleobases that require an activation pathway different from nucleosides to achieve the same active form. We identified some known and previously undescribed dengue virus nucleobase inhibitors and their corresponding nucleosides. Our investigation of the mechanism of action of one nucleobase and its corresponding nucleoside found evidence for enhanced mutagenesis of the dengue virus genome in the presence of the compounds in cell culture. A wide screening of nucleobases libraries is a promising strategy to discover dengue virus inhibitors including potential viral mutagens.

## Introduction

Dengue virus (DENV) is a worldwide health threat, with hundreds of millions of people infected yearly in more than 100 countries [1]. There are four known DENV serotypes and a first infection with one serotype followed by a second infection with another serotype may result in severe disease [2, 3]. For these and other issues, vaccines designed for a pan-serotype protection, including the commercial dengue vaccine approved and used in a few countries, have yielded mixed results [4, 5]. Safety and partial efficacy concerns in addition to cost, storage and delivery issues may hinder implementation of vaccines in many countries.

There are currently no approved drugs to treat DENV infection. Thus far, classical antiviral approaches (e.g. NS5 polymerase inhibitors, entry inhibitors, protease inhibitors, etc.) have yet to provide treatments for DENV infection and therefore the investigation of new antiviral strategies is warranted [6–8]. One such strategy to explore is lethal mutagenesis [9]. The idea of viral lethal mutagenesis is to exploit the natural tendency of RNA viruses to mutate in order to favor the accumulation of deleterious mutations in the newly formed viruses, eventually leading to viral extinction (for review see [10]). DENV and other RNA viruses display a high mutation rate (10^−4^ to 10^−6^ mutations per bp per generation) [11, 12] as an evolutionary characteristic allowing these viruses to escape host immune defense mechanisms and adapt rapidly to new stress conditions [13, 14]. An error-prone viral polymerase combined with a high replication rate are considered to be the main sources of mutations. It is this critical source of viral adaptability (e.g. the virus high mutation rate) that makes RNA viruses a target of choice for antiviral lethal mutagenesis strategies [15–17]. RNA viruses maintain a delicate balance between their need to adapt and their need to preserve a level of genetic integrity put at risk by deleterious mutations [18]. Modifying this fragile equilibrium by increasing the viral mutation rate with mutagens has been proposed as an antiviral strategy [15]. The well-known antiviral nucleoside drug ribavirin induces lethal mutagenesis for different viruses [19–22].

The discovery of new nucleosides as antiviral mutagens has been impaired by several hurdles including the toxicity of the potential drugs as well as synthetic challenges. In addition, potentially mutagenic nucleoside analogues frequently suffer from poor metabolic conversion to the active triphosphate form required by the viral polymerase. The first phosphorylation of the nucleoside analogue is often the rate limiting step to obtain the active mutagenic nucleoside triphosphate used by the viral polymerase [23, 24]. In order to overcome the potential first phosphorylation difficulty, we propose to use nucleobases (purine or pyrimidine base without the ribose or phosphate moieties of a nucleoside). Enzyme mediated condensation of nucleobases with 5-phosphoribosyl-1-pyrophosphate to give the corresponding nucleoside-5’-monophosphate can provide an alternative pathway. Thus, for a nucleoside where the first phosphorylation is inefficient then using its corresponding nucleobase could allow metabolic conversion to the corresponding nucleoside triphosphate, thereby providing a more efficient metabolic conversion to the triphosphate. Studies on nucleobases have been limited [25], with the notable exceptions of 5-fluorouracil and T-705 (favipiravir) [26–32]. However, in the context of targeting viruses that affect developing countries, mutagenic nucleobases present key advantages over mutagenic nucleosides. In addition to their different metabolic activation pathways, nucleobase analogues are considerably cheaper, more diverse and commercially available in higher numbers compared to corresponding nucleoside analogues. The chemical synthesis of a nucleobase is faster and simpler than the synthesis of the corresponding nucleoside. Similarly to nucleosides, nucleobases possess their own cellular transporters [33, 34].

In this study, we describe the identification of five nucleobases and three corresponding nucleosides that possess potent anti-DENV activity. These compounds have not been previously described to have anti-DENV activity, except for the nucleoside **1b** (ribavirin) [35, 36]. We compared the antiviral activities and toxicities of the nucleobases with their corresponding nucleosides. For virus passaged in the presence of a nucleobase **3a** (T-1105) or nucleoside **3b** (T-1106), we detected an increase in mutations compared to virus passaged in DMSO indicating a possible reduction in virus titer via increased mutagenesis. To our knowledge, our study is the first to fully compare the antiviral mechanisms and efficacies of a nucleobase and its corresponding nucleoside, highlighting the differences, similarities and potential advantages of nucleobases versus nucleosides. Our study also highlights the potential of lethal mutagenesis induction during DENV replication as an alternative to classical antiviral strategies.

## Methods

### Compounds

**5a** (T-705) (CAS# 259793-96-9, 6-fluoro-3-hydroxypyrazine-2-carboxamide) was purchased from ASTA Tech. **3a** (T-1105) (CAS# 55321-99-8, 3-Hydroxy-2-pyrazinecarboxamide) was purchased from Alfa Aesar. **3b** (T-1106) was synthesized according to known procedures (Preparation of nucleosides with non-natural bases as anti-viral agents Can. Pat. Appl. (2006), 149pp. CODEN:CPXXEB; CA2600359). **1a** (ribavirin base) (CAS# 3641-08-5, 1,2,4-Triazole-3-carboxamide) was purchased from Ark Pharm. **1b** (ribavirin) (CAS# 36791-04-5) was purchased from Carbosynth. **2a** (mizoribine base) (CAS# 56973-26-3, 5-Hydroxy-1H-imidazole-4-carboxamide) was purchased from Ark Pharm. **2b** (mizoribine) (CAS# 50924-49-7) was purchased from Carbosynth. **4a** (diaminopurine) (CAS# 1904-98-9, 2,6-diaminopurine) was purchased from Sigma-Aldrich. **4b** (diaminopurine riboside) (CAS# 2096-10-8, 2-Aminoadenosine) was purchased from Berry and Associates. **6** (mycophenolic acid) (CAS# 24280-93-1) was purchased from Sigma-Aldrich.

### Cell lines and virus

The hepatocyte-derived cellular carcinoma cell line Huh-7 [37] was used for DENV infection and drug treatment. The African green monkey kidney Vero cell line (ATCC CRL-81) was used to titer DENV via plaque assay. The baby hamster kidney cell line carrying a DENV subgenomic replicon, BHK pD2-hRucPac-2ATG30 [38] (obtained from Dr. M. Diamond, Washington University, School of Medicine), was used for DENV replicon assay. All cell lines were maintained in Dulbecco’s modified Eagle’s (DME) medium supplemented with 10% fetal bovine serum (FBS), 100 IU streptomycin/penicillin per ml and 10 μg/mL plasmocin (InvivoGen) at 37°C in a 5% CO_2_ incubator. DENV replicon cells were supplemented with 3 μg/mL puromycin (Life Technologies). DENV-2 stocks from New Guinea C strain (ATCC VR-1584) were generated from C6/36 mosquito cell cultures (ATCC CRL-1660) grown in Minimum Essential Medium (MEM) supplemented with 10% FBS, 1% non-essential amino acids and 1% sodium pyruvate at 28°C with 5% CO_2_. The C6/36 cells on T-150 flasks were inoculated with virus and the supernatant harvested after complete cytopathic effects. Viral stock titers were determined by plaque assay on Vero cells.

### Cell viability assay

The sensitivity of the cell lines to the compounds was examined using the 3-(4,5-dimethylthiazol-2-yl)-5-(3- carboxymethoxyphenyl)-2-(4-sulfophenyl)-2H-tetrazolium (MTS)-based tetrazolium reduction CellTiter 96 Aqueous Non-Radioactive cell proliferation assay (Promega G5430). The compounds were initially tested at 10 and 50 μM final concentrations. Each plate also contained DMSO alone, medium alone, and an inhibitory compound, **6**. DENV replicon or Huh-7 cells were plated at a density of 1,500 or 8 × 10^3^ cells, respectively, per well in 96-well plates containing 100 μl of culture medium overnight. Compounds were added to triplicate wells in culture medium and incubated for an additional 72 h. MTS reagent was then added to each well and incubated at 37°C in a humidified 5% CO_2_ atmosphere. The plates were read at various time points at a wavelength of 490 nm using a Molecular Devices M5e plate reader. Mean values of triplicate wells were determined and compared to the mean value for the wells that received DMSO alone. For compounds selected for dose-response experiments, the CC_50_ was determined by comparing cell viability for eight serial dilutions of the compound and DMSO treated cells using GraphPad Prism software. The CC_50_ value was defined as the compound concentration resulting in a 50% reduction readout compared with the DMSO.

### DENV replicon assay

Compounds were evaluated for antiviral properties using BHK cells containing a DENV-2 viral replicon. 1.5 × 10^3^ replicon-containing cells per well were plated in white opaque 96-well plates in the absence of antibiotic selection and the next day, compounds dissolved in DMSO were added to triplicate wells in culture medium. The compounds were initially tested at 10 and 50 μM final concentrations and each plate also contained DMSO alone, medium alone, and **6**. Three days later, medium was replaced with a 1:1000 dilution of ViVi-Ren Live Cell Substrate (Promega) in DME minus phenol red and 10% FBS. Luminescence was measured with a Molecular Devices M5e plate reader. Mean values of triplicate wells were determined and compared to the mean value for the wells that received DMSO alone. For compounds selected for dose response experiments, the concentration of compound that reduced luciferase activity by 50% was defined as the 50% effective concentration (EC_50_). The EC_50_ was determined by comparing luciferase activity for eight serial dilutions of the compound and DMSO treated cells using GraphPad Prism software.

### Titer reduction assay

Huh-7 cells were seeded in 12-well plates at a density of 4×10^5^ cells per well in 1 mL culture medium. The next day, cells were washed and inoculated with DENV at a multiplicity of infection (m.o.i.) of 0.2 in 500μl infection medium (MEM containing 2% FBS and 10 mM HEPES). The inoculum was removed after 1h, cells were washed with PBS and then incubated in 1 mL MEM, 2% FBS, 1% pen/strep plus compound for 72 h. Viral supernatants were clarified by centrifugation for 5 min at 1500×g and aliquoted and stored at -80°C. Viral titers were determined using a plaque assay on Vero cells. Briefly, confluent Vero cell monolayers in 24-well plates were incubated at 37°C for 1 h with duplicate 300 μl samples of 10-fold serial dilutions of viral supernatants. The cells were then washed to remove unbound viral particles and overlaid with 500ul MEM containing 1.3% methylcellulose, 5% FBS and 10mM HEPES. After 5 days of incubation at 37°C and 5% CO_2_, cells were washed with PBS, fixed, and stained using 1% Giemsa. Infectious virus titer (pfu/mL) was determined using the following formula: number of plaques × dilution factor × (1/inoculation volume). The viral titer was presented as the mean of duplicate samples from a dilution yielding approximately 20–50 plaques per well.

### Viral RNA extraction, RT-PCR amplification, quantitative PCR, detection of viral genome mutations

For qPCR determination of viral genome copy number, viral RNA was isolated from 140 μL of drug-treated or DMSO-treated infected culture supernatant using QIAamp Viral RNA mini kit (Qiagen), following manufacturer’s protocol. Viral RNA was quantified using the TaqMan RNA-to-C_T_ 1-Step qPCR Kit (Applied Biosystems). Primers used for qPCR were 5’-CATGATGGGAAAAAGAGAGAAGAAGCT-3’ (forward) and 5’-GGCTCTGCTGCCTTTTGC-3’ (reverse) amplifying a region numbering 8928–8988 in the genome (numbering starting from the beginning of genome, accession number KM204118). The qPCR FAM probe sequence is 5’-TTGCCGAACTCCCC-3’. Serial 10-fold dilutions of plasmid containing the NS5 gene of DENV were used to generate a standard curve for the quantification of viral RNA genome copy number based on cycle threshold (*C*_*T*_) values. The limit of detection for NS5 plasmid dilutions was 30 copies (S1 Fig). One-way ANOVA was performed to determine statistical significance of mean genome copy numbers among treatments at each virus passage and Tukey’s honestly significant difference (HSD) used to determine statistical significant (p < 0.05) between DMSO and **3a** or **3b** at each passage. Statistical analysis via GraphPad Prism 5 software. To obtain sequence data from viral RNA isolated at each passage, cDNA was generated via M-MLV reverse transcriptase and random hexamers (New England Biolab Inc.) per manufacturer’s instructions. An approximately 1600-base fragment covering membrane protein (prM) and envelop protein gene (E) of DENV-2 viral genome was amplified using PfuUltra II Fusion HS DNA polymerase (Agilent) with primers prMEfor (5'- AACTCAGAATTCTTCCATTTAACCACACGTAAC-3') and prMErev (5'- AACTCAGAATTCTCCTTTCTTAAACCAGTTGAG -3'). PCR products were purified using Qiaquick PCR purification kit (Qiagen) and then digested with *EcoRI* and ligated into pcDNA3.1 for sequencing. Sequence for approximately 40–50 individual clones per sample was obtained from the University of Minnesota Genomics Center. Sequences were aligned over a 980-base region that had adequate quality sequencing reads for all clones. Only mutations present in both the forward and reverse reads of a clone were counted. All incidence-based determinants of mutation frequency (Mf min, Mf max, Mfe) were calculated as described [39]. A two-tailed Mann-Whitney U test (GraphPad Prism 5 software) was used to determine if there were statistically significant differences for the mean number of mutations per clone between DMSO-treated and each drug-treated virus passage.

### Construction of DENV-2 NS5 plasmid

The DENV-2 viral RNA was isolated from 140 μL of lab stock DENV-2 using QIAamp Viral RNA mini kit (Qiagen). cDNAs corresponding to viral RNAs were generated with random hexamers (New England Biolab Inc.). A 2600-base fragment of NS5 cDNA was amplified using PfuUltra II Fusion HS DNA polymerase (Agilent) with primers NS5for (5'- GGCCAGTGCCAAGCTTGAACTGGCAACATAGGAAGAACGC-3') and NS5Rev (5'- CCGGGGATCCTCTAGACCACAGGACTCCTGCCTCTT -3'). PCR product were purified using Qiaquick PCR purification kit and inserted into a *XbaI* and *HindIII* digested pUC18 vector using In-Fusion HD Cloning Kit (Clontech). A positive clone was identified and the nucleic acid sequence of NS5 confirmed by sequencing at the University of Minnesota Genomics Center.

### Virus and compound passage

Virus was passaged on Huh-7 cells supplemented with 200 μM **3a**, 500 μM **3b** or DMSO (0.5%). Huh-7 cells were seeded in 12-well plates and inoculated with DENV-2 as described for Titer reduction experiments above except an m.o.i. of 0.01 was used. After 3 days of compound treatment, 50 μL of the harvested supernatant was used to inoculate fresh Huh-7 cells in the continued presence of compound. The virus titer in the harvested supernatant was determined by plating ten-fold serial dilutions onto single wells of a 24-well plate. The wells were washed and overlaid as described in the Titer reduction assay above. If no plaques were obtained in any of the harvested supernatant dilutions, the undiluted supernatant was used. If plaques were not detectable, the virus titer was considered to be at the limit of detection, 1 plaque (3.3 pfu/mL). Supernatant titer was determined from a single well where there were approximately 20–50 plaques when possible. This experiment was repeated three times at the compound concentrations listed.

## Results

**Fig 1:**
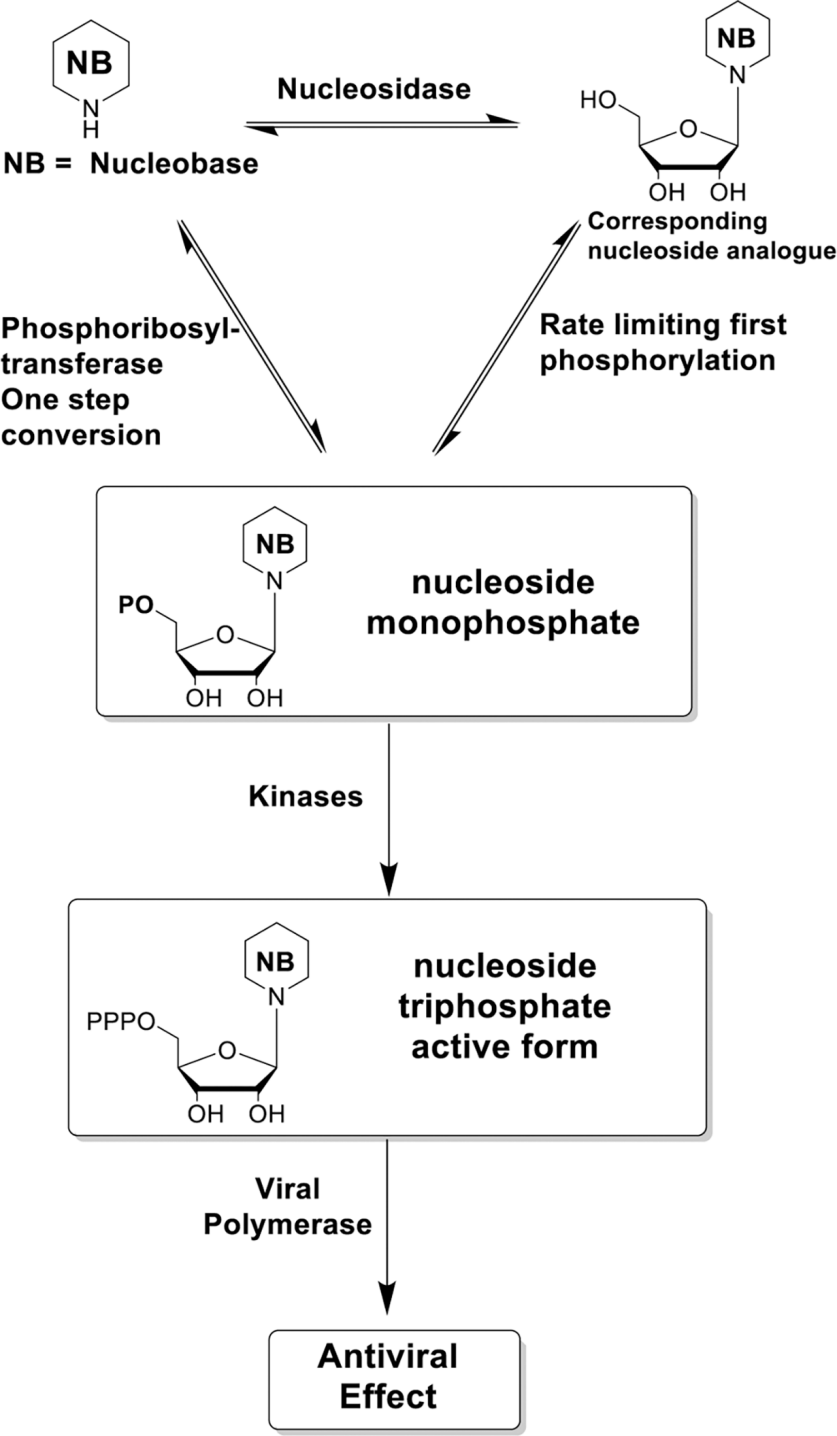
Activation pathways of antiviral nucleobases and nucleosides.

**Fig 2:**
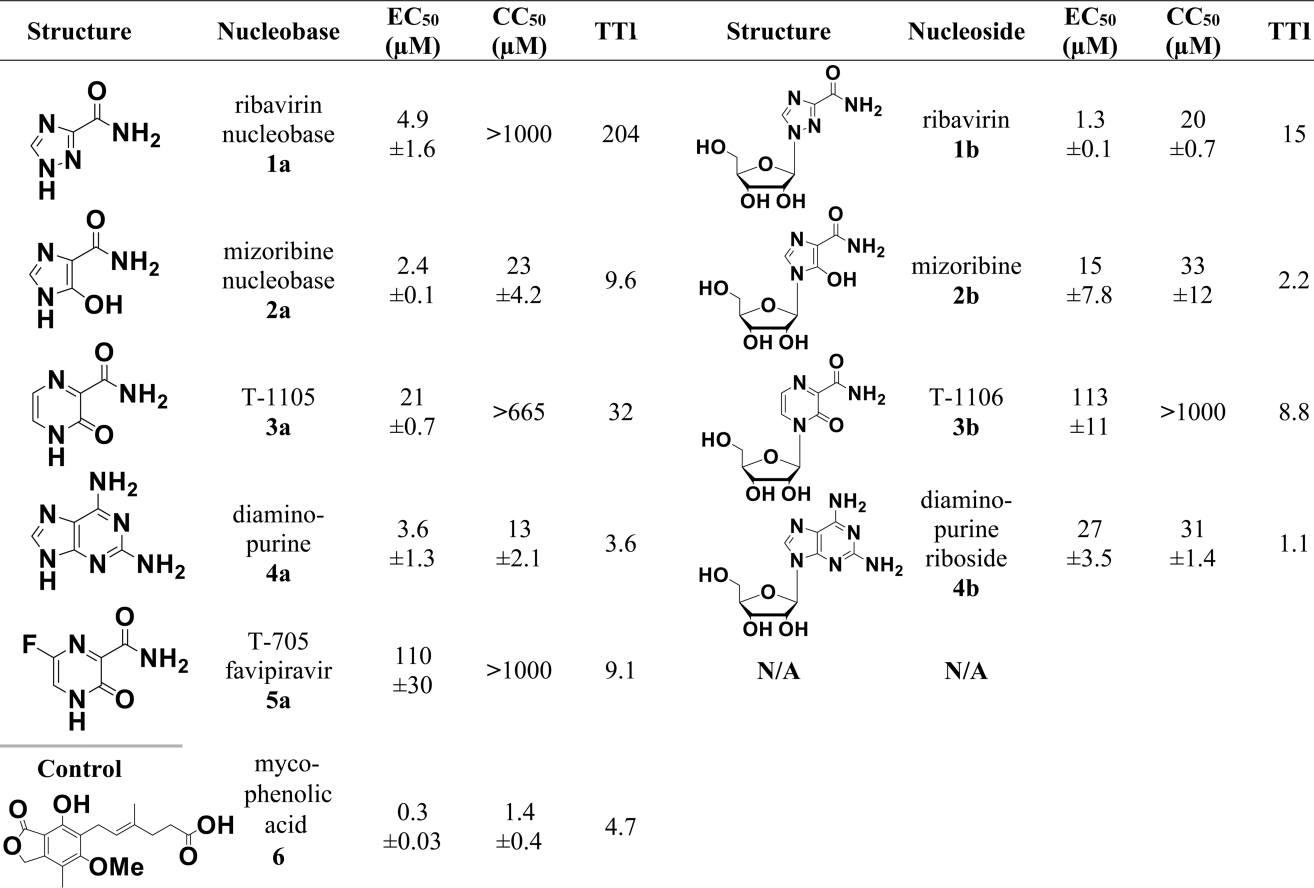
Active nucleobases and their corresponding nucleosides. Dose-response results for selected compounds evaluated in DENV replicon cells. Doses were performed in triplicate and each dose-response experiment was performed independently at least twice. Mean values plus standard deviation for results from each compound are shown. Mycophenolic acid served as a non-nucleobase/nucleoside control inhibitor. TTI is the tissue culture therapeutic index (CC_50_ / EC_50_). N/A = not available.

Antiviral nucleoside identification can be hindered by difficulties in chemical synthesis and poor conversion of the nucleoside to the active triphosphate form. To circumvent these issues, we propose to use nucleobases in our initial screen for antiviral agents because of their different activation pathway to the active nucleotide (Fig 1), their low cost and ready commercial availability. Phosphoribosyl transferases of the cellular nucleotide salvage pathway directly convert some nucleobases to the corresponding nucleoside monophosphate and therefore the corresponding nucleoside analogue need not be an efficient substrate for a nucleoside kinase (Fig 1) [40, 41]. In that regard, **3a** and analogue **5a** (Fig 2) are substrates of human phosphoribosyl transferases and are converted in one step to the corresponding nucleoside monophosphate [41].

**Fig 3:**
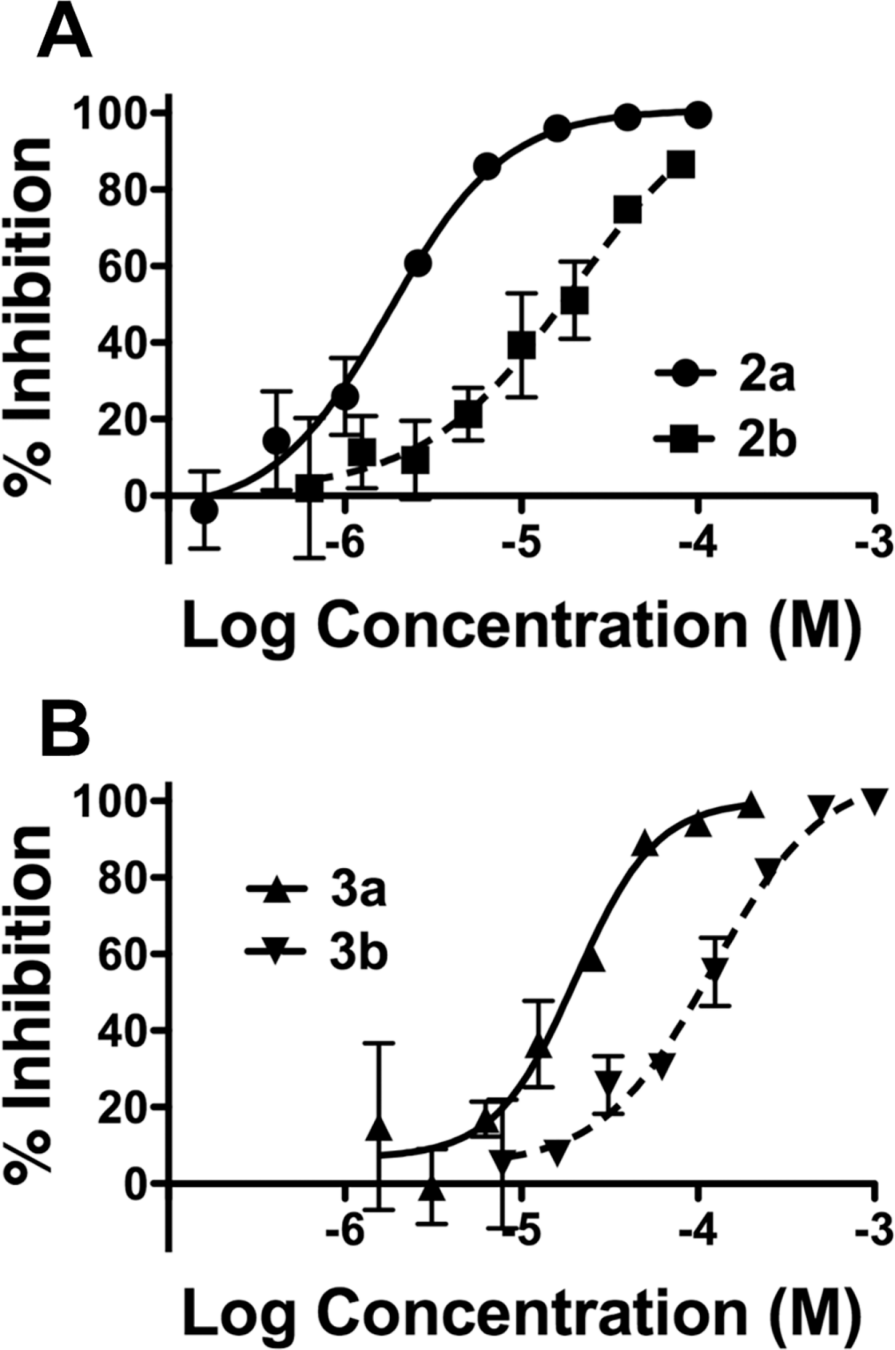
Dose-response curves for nucleosides and nucleobases. The percent (%) inhibition of each compound over the log_10_ of concentrations compared to DMSO alone is shown. Doses were performed in triplicate and repeated at least twice. Average values plus standard deviation for each dose for one representative experiment are shown. Error bars in **A** and **B** may be too small to be seen.

Our strategy to identify nucleobase and nucleoside DENV inhibitors was to screen for activity and toxicity of selected compounds at 10 μM and 50 μM using a luciferase-reporting DENV replicon cell line, BHK pD2-hRucPac-2ATG30 [38]. Compounds that demonstrated inhibitory activity against the replicon cell line were used in dose-response analysis to assign EC_50_ and CC_50_ values. The nucleobases were generally more active with a higher tissue culture therapeutic index (CC_50_/EC_50_) than their corresponding nucleosides (Fig 2). The EC_50_ values of the active nucleobases range from 2.4 to 110μM, comparable to the EC_50_ values of the active nucleosides that range from 1.3 to 113μM (Fig 2). Nucleobase **3a** is 5 times more active than nucleobase **5a** (favipiravir). The CC_50_ values of the nucleobases **1a**, **3a** and **5a** were beyond 665μM (Fig 2). Remarkably, nucleobase **1a** did not show cytotoxicity at 1000 μM compared to **1b** nucleoside (Fig 2) where the CC_50_ was 20 μM. **2a** nucleobase displayed a clear antiviral effect at 2.4 μM. Representative EC_50_ curves for **2a** plus corresponding nucleoside **2b** and for **3a** plus corresponding nucleoside **3b** are shown in Fig 3. Inactive nucleobases at initial screening are listed in S1 Table in the supplementary material.

We focused on the antiviral mechanism of nucleobase **3a** and corresponding nucleoside **3b** for the following reasons: 1) among the 5 active nucleobases, **5a** was already known to induce viral lethal mutagenesis but its corresponding nucleoside was not available and unable to be synthesized for our study; 2) **1a** and **2a** are likely to possess a complex mechanism of action due to their probable inhibitory effect of inosine monophosphate dehydrogenase (IMPDH) after conversion to the nucleotide form; 3) **4b** was too toxic to perform a full study comparing the nucleobase to the nucleoside (Fig 2). Therefore, we chose **3a**, known to be substrate of human phosphoribosyl transferase [41], and its corresponding nucleoside **3b** to be the best candidates for antiviral mechanism of action studies and to compare the effects of nucleobase and corresponding nucleoside.

**Fig 4:**
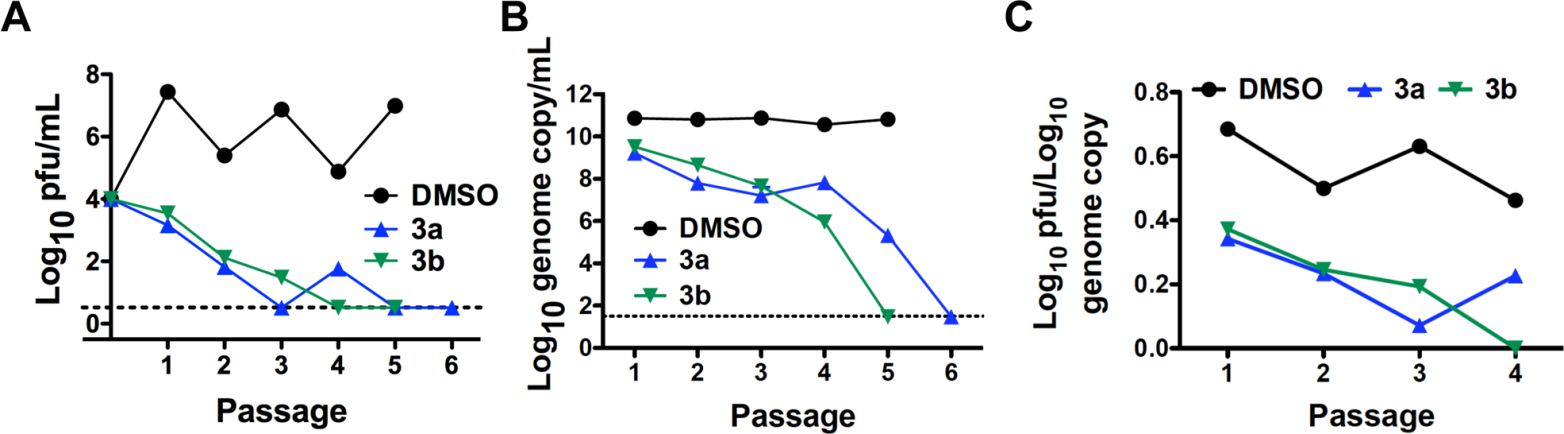
Anti-DENV effects during serial passage of virus in the presence of antiviral nucleobase and nucleosides. Virus passaged in Huh-7 cells in the presence of **3a** (200 μM, blue) and **3b** (500 μM, green). Cells were initially inoculated at an m.o.i. of 0.01 and cultured in compound at the indicated concentration. Every 72 hours a fixed volume of supernatant was used to inoculate fresh cells maintained in compound. **A**. The titer of virus in the supernatant was obtained by serial dilution and counting plaques in single dilution containing 20–50 plaques when possible (see Methods). This experiment was repeated three times at these compound concentrations with similar results and a representative experiment is shown. The dashed line indicates the limit of detection of 3.3 pfu/mL (see Methods). **B**. Genome copy equivalents were monitored for the passages indicated using RT-qPCR. Mean values and standard deviations of duplicate measurements are shown. All points have standard deviations represented by error bars however some error bars may be too small to be seen. This experiment was performed twice with similar results. The dashed line indicates the limit of detection of 30 copies of NS5 gene (see Methods). **C**. Specific infectivity was calculated as log_10_ titer divided by log_10_ genome copy equivalents for each passage and treatment.

Our approach to study mechanism of action and possible lethal mutagenesis was to passage virus in Huh-7 cells in the presence of a compound and determine the compound’s effect on virus titer, genome copy number and genome sequence. We had initially identified inhibitors using a DENV replicon BHK cell line so we wanted to verify inhibitory activity in Huh-7 cells using replication competent DENV. We chose Huh-7 cells because they are a human cell line, they are susceptible to DENV infection and they produce readily detectable infectious virus. The BHK replicon cells are a convenient tool to identify initial DENV inhibitors. We used human cells for more detailed studies for these compounds that require conversion to the active form by cellular enzymes. We repeated the dose-response experiments for **3a** and **3b** using a titer-reduction assay with Huh-7 cells as previously described [42]. The values obtained for the compounds (Table 1) were consistent with those from the replicon assay. Based upon the data in Table 1, we used non-toxic levels of **3a** (200 μM) and **3b** (500 μM) that we empirically determined (S2 Fig) would reduce virus replication steadily during multiple passages and ultimately lead to undetectable levels of infectious virus. The results for virus passage in the presence of **3a** (200 μM), **3b** (500 μM) or DMSO (0.5%) are shown in Fig 4. Compounds were added to Huh-7 cells shortly after inoculation. After 3 days incubation in compound, the supernatant was collected and a fixed volume (50 μL) of supernatant was used to inoculate fresh cells. The level of infectious virus present in the supernatant for each passage was determined by plaque assay and the number of viral genomes by RT-qPCR. For the RT-qPCR, we chose primers within the NS5 gene to increase likelihood of detecting full-length genomes. Both compounds induced a steady decline in infectious virus production and supernatant genome copy number over repeated passages (Fig 4). In the presence of **3b**, infectious virus was undetectable at passages 4 and 5 and genomic RNA was undetectable at passage 5 (Fig 4). For **3a**, no infectious virus was detected at passages 5 and 6 and no genomic RNA was detected at passage 6 (Fig 4). The mean genome copy numbers (Fig 4B) for the three treatments were statistically different at each passage (p < 0.001, One Way ANOVA) with statistically significant differences between **3a** and DMSO and between **3b** and DMSO at each passage (p < 0.05, Tukey’s HSD). Both compounds displayed a reduction in the ratio of infectious virus to RNA genome copy number (sometimes referred to as RNA specific infectivity) when compared to DMSO (Fig 4). This delay in reduction of genomic RNA compared to infectious virus is a hallmark of a mutagenesis-based antiviral activity [43–46]. These observations, along with the knowledge that **5a** (analogue of **3a** and **3b**) induces mutagenesis in influenza A, norovirus and the flavivirus West Nile virus [27–29], motivated us to determine if a mechanism of action for **3a** or **3b** included an enhanced mutagenesis of the viral genome.

**Table 1:** Titer reduction assay dose-response.

Compound	EC_50_ (μM)	CC_50_ (μM)
**3a**	20 ± 11	>1000
**3b**	60 ± 22	>1000

We hypothesized that an increase in mutations induced by a particular nucleobase or nucleoside would be detected by analyzing the viral genome sequence at a passage near where the titer was significantly reduced or undetectable. Therefore, we amplified a region containing the pre-membrane (prM) and envelope (E) genes from viral genomic cDNA derived from passage 3 **3b**-treated cells and passage 4 **3a**-treated cells because those passages were just before viral titer was undetectable. We amplified the prM/E region of the viral RNAs instead of the downstream NS5 gene to increase the likelihood we would obtain PCR products when viral titers were greatly reduced. The presence of the NS5 gene would require an almost complete genomic RNA whereas the prM/E region was closer to the 5’ end of the viral genomic RNA its presence would not require a complete viral genome. The amplified products were inserted into a cloning vector and the nucleotide sequence was analyzed for the resulting 35–50 independent clones. We sequenced at least 31,000 nucleotides for each set of clones similar to other published studies of viral lethal mutagenesis [27, 43] and compared the sequences to that of the consensus sequence obtained from DMSO-treated cell supernatants at passages 3 and 4.

**Fig 5:**
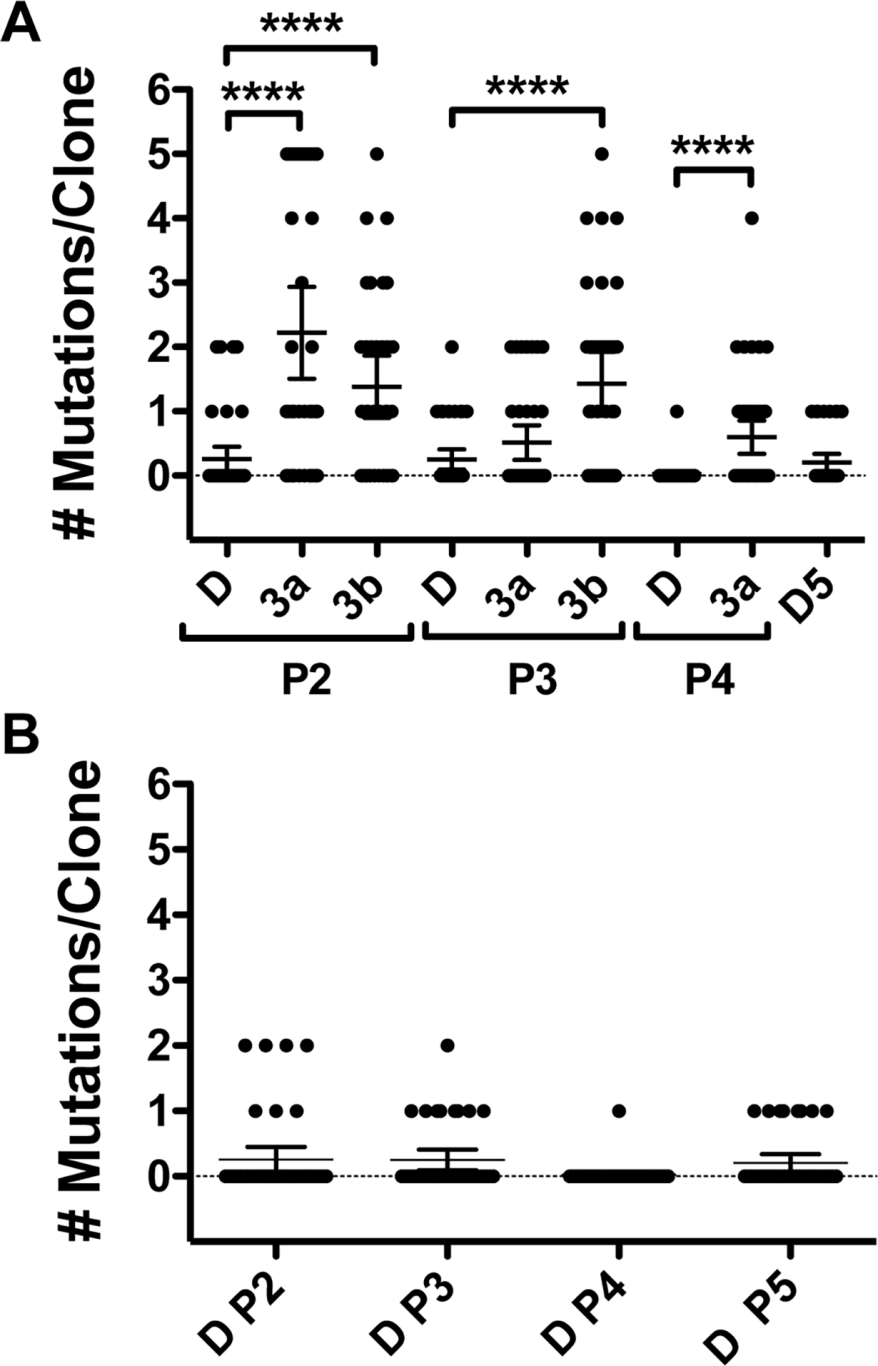
The number of mutations per sequence clone. **A**. Virus was passaged in cells treated with compounds indicated. The prM/E region from viral RNA was amplified using RT-PCR, ligated into a vector and approximately 35–50 independent clones were chosen for each drug treatment at indicated passage. The nucleotide sequence of the DENV region was determined using standard Sanger sequencing methods for each clone. Thin black horizontal bar indicates the mean number of mutations per clone and the vertical bars indicate the 95% confidence intervals. Four asterisks signify p < 0.0001. P values determined using a two-tailed Mann-Whitney U test. **B**. The DMSO passage data from **A** graphed separately. P = passage and D = DMSO.

An initial plot and analysis of the number of mutations per clone for **3a** treatment passage 4 and **3b** passage 3 clearly indicate there was a significant difference (p < 0.0001) in the number of mutations per clone for sequence obtained from compound-passaged virus compared to DMSO (Fig 5A). We next calculated a number of incidence-based mutation indices to better determine possible heightened mutagenesis as recommended in a recent review [39]. There was an increase in every index of mutation frequency and sequence diversity for **3a**- and **3b**-treated clones compared to those for DMSO (Table 2). These include, the minimum mutation frequency (Mf min, mutation at a given nucleotide counted only once), maximum mutation frequency (Mf max, all mutations counted) and the entity level mutation frequency (Mfe, mutations with respect to sequence of dominant haplotype of drug-treated clones). In addition, the number of haplotypes, number of different mutations, number of total mutations and number of clones with a mutation (Table 2) were all higher for **3a** and **3b** passage sequences. We noted fewer mutations in the DMSO passage 4 sequence than seen for other DMSO passages (Table 2 and Fig 5B). That does not change our interpretation of the **3a** passage 4 results. The mean number of mutations per clone for **3a** passage 4 was significantly higher than the mean mutations per clone for any DMSO passage (ranging from p < 0.01 to p < 0.03). In addition, all advanced indices of mutation frequency and sequence diversity were greater for **3a** passage 4 when compared to all DMSO passages (Table 2). Lastly, we examined sequence data from other **3a-** and **3b**-treated passages. Sequence from passage 2 also showed higher numbers of mutations per clone and an increase in all advanced mutation indices for **3a**- and **3b**-passaged virus compared to DMSO (Fig 5 and Table 2). The sequence data from **3a** passage 3 yielded mixed results with certain measures of mutation frequency similar to DMSO and certain others higher than those for DMSO (Table 2 and Fig 5A). We were unable to obtain sequence data for **3b** passage 4 or 5. Taken together, these results indicate an enhanced mutagenesis as a likely significant contributor to the antiviral mechanism of action for **3a** and **3b**.

**Table 2:** Sequence analysis of DENV passages.

Passage (P)		P 2			P 3			P 4		P 5
		DMSO	3a^b^	3b^b^	DMSO	3a	3b	DMSO	3a	DMSO
Transition	G→A		3	6	1	3	1		3	
	A→G	2	3	6	1		4	1	2	
	U→C		1	14	4	2	3		4	4
	C→U	2	4	4	1	3	6		3	2
Transversion	U→A	1	2				1		2	
	G→U	1	2	2		1				
	U→G	1	1	1						
	C→A								2	1
	A→U		1							
Insertion					1					
Deletion					1					
Number of different mutations		7	17	33	9	9	15	1	16	7
Number of total mutations		11	80	47	10	19	50	1	27	8
Number of nucleotides sequenced		38,829	32,508	30,702	36,120	33,411	31,605	40,635	40,635	35,217
Number of haplotypes		7	17	22	9	9	17	2	15	8
Number of clones with mutation/total		7/43	26/36	22/34	9/40	12/37	22/35	1/45	19/45	8/39
Mf min^a^, per 10,000 nucleotides		1.8	5.2	10.7	2.5	2.7	4.7	0.2	3.9	2.0
Mf max^a^, per 10,000 nucleotides		2.8	24.6	15.3	2.8	5.7	15.8	0.2	6.6	2.3
Mfe^a^ (×10^−3^)		1.4	2.5	2.4	1.1	1.4	2.2	0.6	1.6	1.0

^a^Defined in the text

^b^**3a** 200 μM and **3b** 500 μM for all passages

In the sequence analysis, we excluded nucleotides 952–4 (numbering starting from the beginning of genome, accession number KM204118) in analysis of mutations. This codon, located in the E gene, changes in response to repeated DENV passaging in cultured cells [47, 48]. The sequence for nucleotides 952–4 of our stock virus was ATA. However, nearly all the clones from virus passaged with DMSO, **3a** and **3b** displayed changes in that sequence (S2 Table). We also excluded mutations that were present in a majority of clones and therefore were consensus sequence changes. These included one nucleotide position (1043) in the **3a** clones and three positions (960, 1057 and 1493) in the **3b** clones (S2 Table). A hallmark of lethal mutagenesis is an unchanged consensus sequence despite an increase in single mutations [44, 45] and so it is possible those changes were introduced during the RT-PCR amplification of the viral RNA genomes.

An examination of the types of mutations induced by nucleoside **3b** revealed an almost exclusive increase in transition mutations for purines and pyrimidines (Table 2). For nucleobase **3a**, a similar increase in mostly transition mutations was observed (Table 2). These results are also consistent with data observed with related compound **5a** [27, 28].

## Discussion

Effective and inexpensive drug therapies for DENV infection are urgently needed. So far, classical antiviral strategies have failed to identify small molecules to treat DENV infection. In addition, developing broad antiviral strategies is critical to face future outbreaks of emerging viruses. The adaptability of viruses to changing environments results in the generation of a heterogeneous population of closely related yet different viral variants during infection. The diversity present within this population may result in viruses escaping drug treatments or host immune defenses. Lethal mutagenesis aims to generate deleterious viral mutations that would prevent viral adaptation and drive the viral population to collapse. Induction of lethal mutagenesis has largely been based on using nucleoside mutagens to target the error prone viral polymerase, a main source of viral mutations. However, limitations in the chemical synthesis of nucleosides and poor metabolism to the active triphosphate form have limited the discovery of new viral mutagens. Here, we have identified nucleobases and nucleosides with significant antiviral activity against DENV, some of which appear to act through lethal mutagenesis. Along with nucleosides, nucleobases may be valuable molecules to induce viral lethal mutagenesis or other antiviral effects. For DENV antiviral therapy, nucleobases are cheaper and display a better therapeutic index in cell culture compared to nucleosides. This result warrants further exploration. Overall, our study supports antiviral lethal mutagenesis as a potentially effective strategy to target DENV.

In this study, we have identified five nucleobases and three nucleosides that possess anti-DENV activity. Nucleobase **3a** and corresponding nucleoside **3b** were selected for antiviral mechanism-of-action studies. **3a** and **3b** both increased mutations. Although the antiviral mechanisms of **5a**, **1b** and **4b** have not been explored in this study, these compounds are known antiviral mutagens [22, 25, 28] supporting our result that viral lethal mutagenesis may be an effective antiviral strategy against DENV. The strong antiviral effects of **2b** (EC_50_ 15μM) and its nucleobase **2a** (EC_50_ 2.4μM) suggest a critical role of IMPDH for DENV replication, yet the fact that **2a** and **2b** possess a rotatable amide bond (theoretically able to mimic either adenosine or guanosine) indicates a component of the antiviral effect could occur through lethal mutagenesis. To our knowledge, this is the first description of **2a** as an antiviral agent.

**Fig 6:**
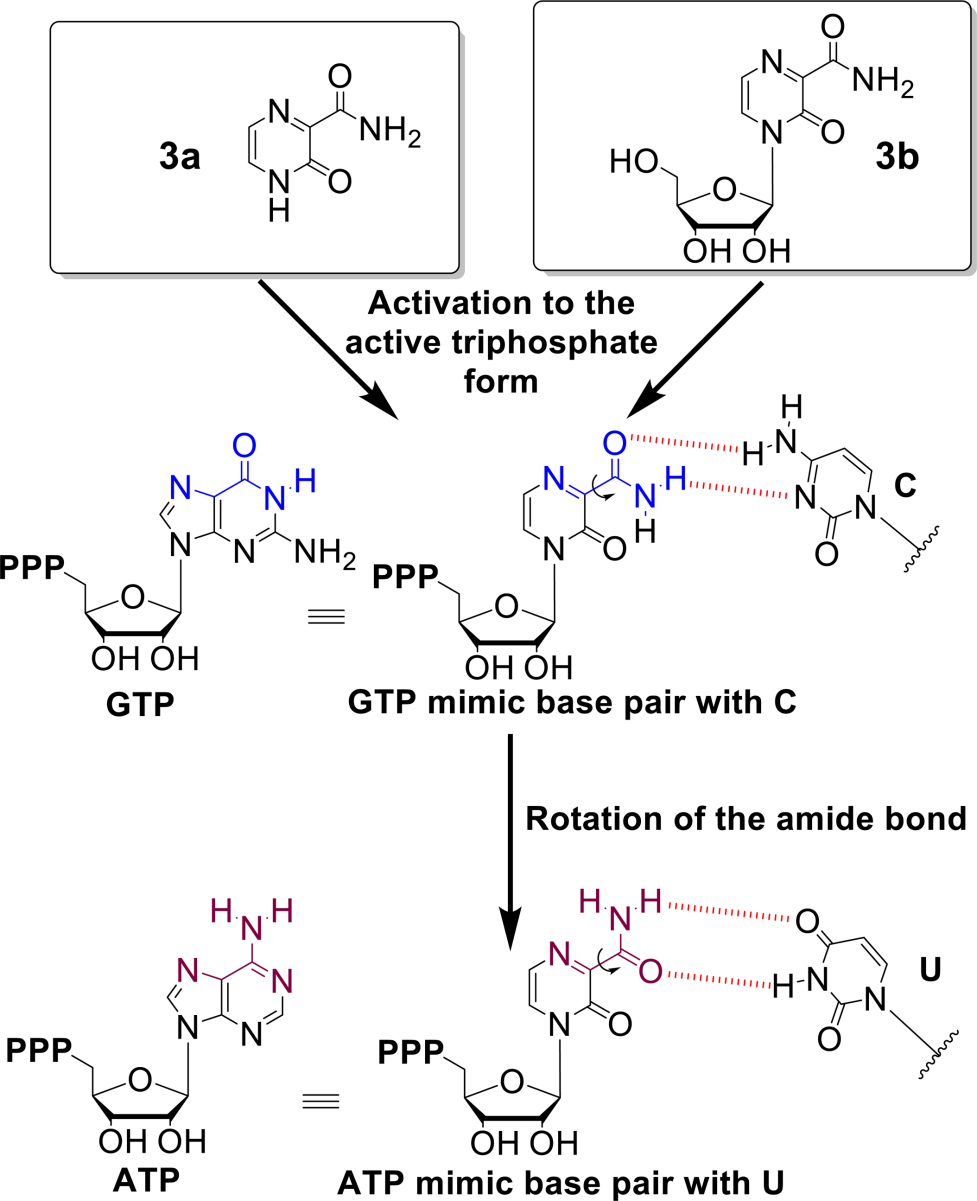
Potential base pairing of nucleoside triphosphate forms of 3a and 3b. Once converted to the active nucleoside triphosphate forms, mutagens **3a** and **3b** may adopt different conformations due to the rotation of the amide bond allowing these compounds to mimic GTP and base pair with C or mimic ATP and base pair with U.

Interestingly, in our study, the active nucleobases and nucleosides (Fig 2) share common structural characteristics such as 1) a purine analog structure and/or 2) a rotatable amide bond. Some of these nucleobases have been described to be converted metabolically to the active nucleotide form [41]. Our study is the first to compare a nucleobase side by side with its corresponding nucleoside for their abilities to induce viral genome mutagenesis as an antiviral mechanism of action. The way by which mutagenic nucleobases/nucleosides and resulting mutagenic nucleotides induce mutations is usually due to ambiguous base pairing misinterpreted by the viral polymerase when replicating the viral genome [49]. Typically, ambiguous base pairing originates from rotational or tautomeric forms of the base that result in the mutagenic nucleoside triphosphate resembling more than one natural nucleoside triphosphate. In that matter, **5a**, an analogue of **3a** and **3b**, was found to be converted to the active triphosphate form and then used by influenza polymerase as an A or G mimic, likely by rotation of its amide bond [50]. In a similar fashion, **1b** (ribavirin) possesses a rotatable amide bond and is widely accepted as an A or G mimic. Overall, **3a** and **3b** in this study and **5a** and **1b** in previous studies all essentially induce transition mutations [22, 27], a result compatible with these molecules resembling A or G. The **3b** triphosphate would manifest as an ATP or GTP mimic and the rotation of its amide bond would allow base pairing with a U or C, respectively (Fig 6). We did not observe a clear preference for A to G and U to C transition mutations versus the G to A and C to U mutations indicating that the DENV polymerase may not have a preference for using **3b** triphosphate. The proportions of A to G or G to A transitions induced by these mutagens can vary from virus to virus [27, 28]. The affinity of the different viral polymerases for one conformation of a mutagen rather than another may explain the observed variance. A mutagen such as **1b** also inhibits IMPDH, an enzyme involved in nucleotide biosynthesis, resulting in an imbalance in endogenous nucleotide pools which may impact the induced mutation spectrum compared to a purely mutagenic compound. In our case, we did not observe major trends for one particular transition mutation versus another. Exploring molecules capable of inducing transversion mutations may be of interest to induce a stronger antiviral effect.

## Supporting information

S1 Table Inactive molecules. (PDF)

S2 Table List of sequence changes for prM/E region cDNA. (XLSX)

S1 Fig Standard curve for DENV NS5 qPCR. (PDF)

S2 Fig Passage of virus in different concentrations of 3a and 3b. (PDF)
