# Supplementary material for: Nucleobases and corresponding nucleosides display potent antiviral activities against dengue virus possibly through viral lethal mutagenesis
Source: PLoS Negl Trop Dis. 2018 Apr 19;12(4):e0006421. doi: 10.1371/journal.pntd.0006421 (PMC5929572; doi:10.1371/journal.pntd.0006421)
Supplement: S1 Table — (PDF) [file pntd.0006421.s001.pdf]

S1 Table. Inactive molecules

Entry	Structure	Entry	Structure	Entry	Structure
**7a**	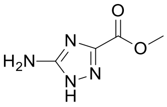	**16a**	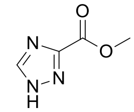	**25a**	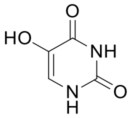
**8a**	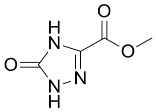	**17a**	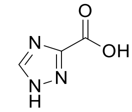		
**9a**	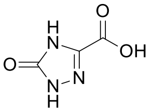	**18a**	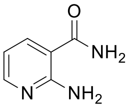		
**10a**	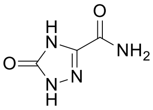	**19a**	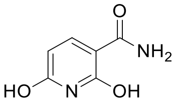		
**11a**	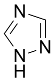	**20a**	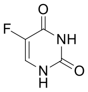		
**12a**	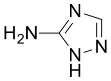	**21a**	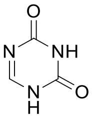		
**13a**	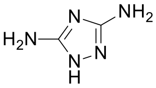	**22a**	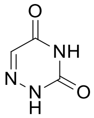		
**14a**	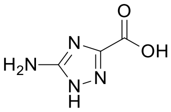	**23a**	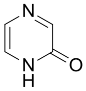		
**15a**	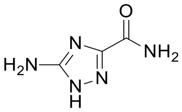	**24a**	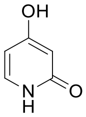		
