# Supplementary material for: Nucleobases and corresponding nucleosides display potent antiviral activities against dengue virus possibly through viral lethal mutagenesis
Source: PLoS Negl Trop Dis. 2018 Apr 19;12(4):e0006421. doi: 10.1371/journal.pntd.0006421 (PMC5929572; doi:10.1371/journal.pntd.0006421)
Supplement: S1 Fig — (PDF) [file pntd.0006421.s003.pdf]

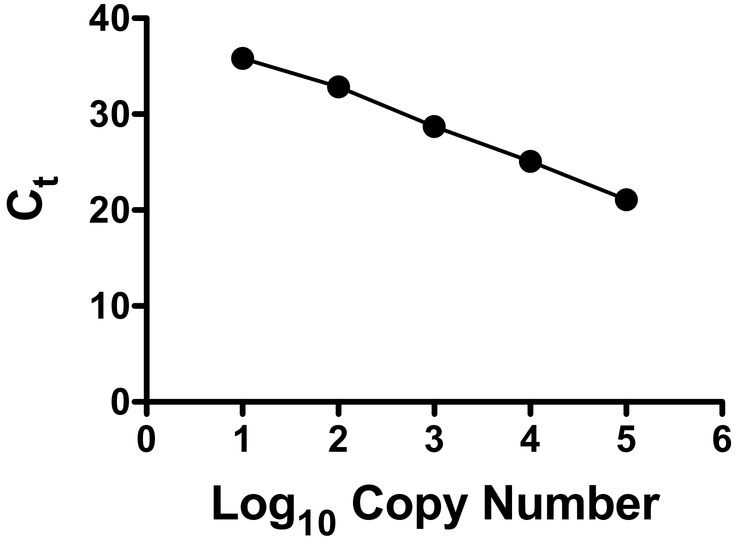

S1 Figure. **Standard curve for DENV NS5 qPCR**. Serial ten-fold dilutions of a plasmid containing the DENV NS5 cDNA (range 30-300,000 copies) were analyzed by qPCR as described in the Methods. Each dilution was performed in duplicate and mean value plus standard deviation of C_t was plotted. The error bars may be too small to be seen. Thirty copies of plasmid was the smallest number for which we could detect a reliable $C_{t,}$ six plasmid copies yielded no C_t value (not available or n/a). $R^2 = 0.998$. The negative control (no plasmid) yielded no C_t values. This experiment was repeated two independent times.
