# Supplementary material for: Nucleobases and corresponding nucleosides display potent antiviral activities against dengue virus possibly through viral lethal mutagenesis
Source: PLoS Negl Trop Dis. 2018 Apr 19;12(4):e0006421. doi: 10.1371/journal.pntd.0006421 (PMC5929572; doi:10.1371/journal.pntd.0006421)
Supplement: S2 Fig — (PDF) [file pntd.0006421.s004.pdf]

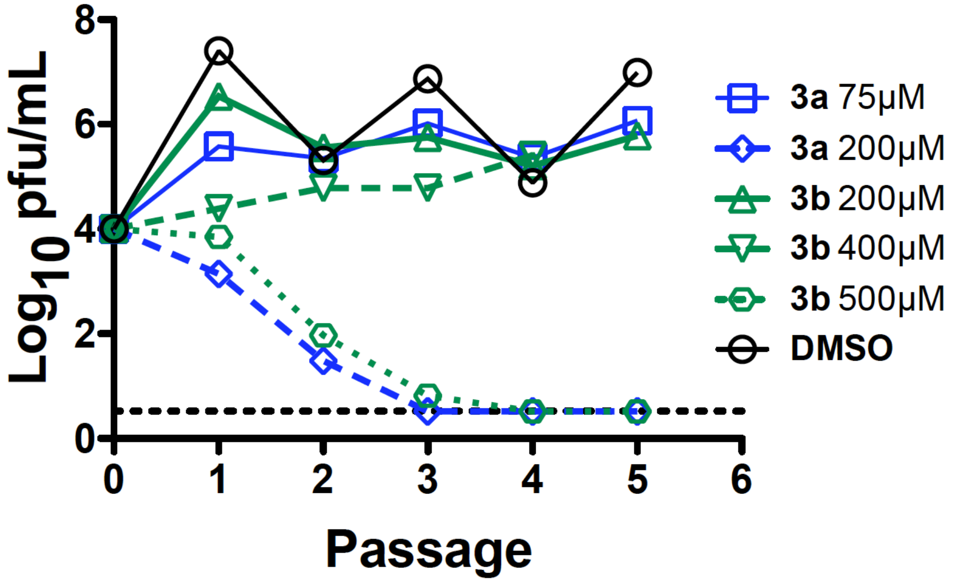

S2 Figure. Passage of virus in different concentrations of 3a and 3b. Huh-7 cells inoculated at an m.o.i. of 0.01 and cultured in compound at the indicated concentration. Every 72 hours a fixed volume of supernatant was used to inoculate fresh cells maintained in compound. The titer of virus in the supernatant was obtained by serial dilution and counting plaques in single dilution containing 20-50 plaques when possible (see Methods). The dashed line indicates the limit of detection of 3.3 pfu/mL (see Methods). Final percentage DMSO = 0.5%.
